# Are molecular tests necessary to diagnose NIFTP? 

**DOI:** 10.18632/genesandcancer.213

**Published:** 2021-03-15

**Authors:** Artur Kuchareczko, Janusz Kopczyński, Artur Kowalik, Kinga Hińcza, Agnieszka Płusa, Stanisław Góźdź, Aldona Kowalska

**Affiliations:** ^1^Endocrinology Clinic of Holycross Cancer Centre, Kielce, Poland; ^2^Department of Pathology, Holycross Cancer Centre, Kielce, Poland; ^3^Molecular Diagnostics, Holycross Cancer Centre, Kielce, Poland; ^4^Division of Medical Biology, Institute of Biology, Jan Kochanowski University, Kielce, Poland; ^5^Collegium Medicum, Jan Kochanowski University, Kielce, Poland; ^6^Department of Clinical Oncology, Holycross Cancer Centre, Kielce, Poland

**Keywords:** NIFTP, BRAFV600E, papillae, cancer, neoplasm

## Abstract

In 2016, encapsulated follicular variant of papillary thyroid carcinoma (EFVPTC) was reclassified as noninvasive follicular thyroid neoplasm with papillary-like nuclear features (NIFTP). In 2018 the criteria for NIFTP were widened by the inclusion of the complete lack of papillae. Secondary criteria, which include molecular examination, are helpful but not required for NIFTP diagnose.

The aim of this study was to assess the molecular background of NIFTP and to answer the question if the aplication of revised criteria for NIFTP diagnosis is associated with the lack of oncogenic mutation.

Repeat histopathological assessment of 1117 cases of papillary thyroid carcinoma (PTC) from 2000-2016 was conducted. Using initial (2016) and revised (2018) diagnostic criteria, NIFTP was diagnosed in 23 and 13 patients respectively. 50 tumor genes hotspots mutation analysis was conducted. BRAF^*V600E*^ mutations were detected in patients who fulfilled only initial NIFTP criteria. Other high-risk mutations (*TP53*) were found in both groups of patients.

The application of restrictive, revised diagnostic criteria for NIFTP negates the need for BRAF^*V600E*^ examination, but these tumors still can harbor other high-risk oncogenic mutations nonetheless. Thus, molecular examination should be considered as a necessary step in NIFTP diagnostic process.

## INTRODUCTION

Worldwide, increasing numbers of people are developing cancer, including cancer of the thyroid gland [1]. An increase in the diagnosis of thyroid cancer has resulted from the introduction of more precise diagnostic methods and wider access to them, and is associated with over-diagnosis [2]. It is estimated that, over the last 30 years in the United States alone, more than twice as many new cases of thyroid cancer have been diagnosed, primarily tumors < 2 cm in size [3]. Despite the increase in cases of thyroid cancer, the 10-year overall survival rate for papillary thyroid carcinoma (PTC) is approximately 97% [4]. One histopathological variant of PTC, with indolent behavior, is encapsulated follicular variant PTC (EFVPTC), which has been re-classified as “non-invasive follicular thyroid neoplasm with papillary-like nuclear features” (NIFTP), based on recommendations from medical experts [5, 6]. 

In the 2017 World Health Organization classification of neoplasms, NIFTP was removed from the list of cancers, emphasizing its very good treatment outcomes and the mild course of the disease in the follow-up period due to its very limited malignant potential [5, 6, 7]. The American Thyroid Association (ATA) also accepted the suggested change and included limitation of aggressive treatments in its recommendations, relative to the treatment of thyroid neoplasms (scope of operation, lobectomy, without 131 I treatment or suppressive doses of L-T4) [8]. However, there is still no consensus how NIFTP cases should be monitored [8]. Periodic monitoring based on serum thyroglobulin levels and neck ultrasound should be considered for patients with NIFTP, but long-term results of this kind of approach are still the subject of ongoing research and more data for more precise recommendation regarding follow-up procedures are needed [8]. 

Initially, NIFTP was thought to potentially constitute 15–20% of all PTC variants. The changed classification meant that a large group of patients would avoid being stigmatized by a cancer diagnosis and would not receive aggressive treatment; however, the initial data were significantly over-stated, and did not include research in Asian populations [9]. Currently, the NIFTP variant is estimated to constitute 9.1% of all PTC cancers (1.6% in Asian populations and 13.3% in western populations) [9].

Consideration of changes to the NIFTP diagnostic criteria, including elimination of all cases with any number of normal papillae could mean that the actual percentage of NIFTP cases is even smaller. Secondary criteria are helpful, but not required for NIFTP diagnose, include molecular examination of BRAF*^V600E^* and other typical high-risk oncogenic mutations (the TERT promoter mutation, *TP53* mutation) [10]. Of note, the use of molecular examination in addition to the histopathological tests is not standard procedure, and most pathomorphological laboratories do not have the equipment necessary to conduct molecular analyses. 

The aim of this study was to assess the prevalence of NIFTP cases in a population of Polish patients with PTC using both the original and revised criteria, together with the molecular background, to determine whether it is necessary to apply full-panel molecular diagnostic techniques where the revised histological criteria for NIFTP are strictly applied. 

## RESULTS

Data on the clinical and pathomorphological features of patients diagnosed with PTC, including cancer stage according to the 8th Edition on American Joint Committee on Cancer Staging (AJCC 8th Edition), risk stratification for cancer recurrence and response to treatment according to 2015 American Thyroid Association Management Guidelines for Adult Patients with Thyroid Nodules and Differentiated Thyroid Cancer (ATA), are presented in Table 1 [11, 12]. 

Despite the increasing rate of FVPTC (follicular variant PTC) in recent years, only 15,5% of all PTC in our study were identified as the FVPTC subtype [13]. The similar rate of FVPTC can be observed in some older studies, with a high number of PTC cases combined with long-term follow-up [14]. 

This might partially result from the inclusion of those patients in our study, who were operated on nearly 20 years ago. The aim of that was the assessment of the long-term response to the treatment applied. 

Of 173 histopathology reviewed PTC follicular subtype cases, 26 tumors were classified as potential NIFTP. After examination by two pathologists 3 cases were excluded from this group based on NIFTP 2016 criteria. There were 2 cases of solid growth >30% and one case of psammoma bodies. 

NIFTP was diagnosed using the 2016 criteria in 23 patients, constituting 2.06% of all patients diagnosed with PTC, while it was diagnosed in 13 patients (1.16% of those diagnosed with PTC) using the revised 2018 criteria. 

All 23 cases of NIFTP were encapsulated. There were no cases of clear demarcation NIFTP. All 23 cases had nuclear score 3. All tumors were examined entirely. None of the 13 cases diagnosed using the 2018 criteria had BRAF^*V600E*^ or *TERT* promoter mutations. There were 3 cases of *TP53* mutation with one case of concurrent HRAS and TP53 mutation in this group. All 13 cases showed a complete lack of papillae on repeated histopathological examination. The characteristics of patients with NIFTP are presented in Table 2. 

Using the revised criteria, 10 patients did not qualify for diagnosis with NIFTP because <1% papillae were detected. Moreover, five of these patients (50%) had the BRAF*^V600E^* mutation. There was one case (10%) of *TP53* mutation in this group. A single papillae was spotted in all those cases. The characteristics of excluded patients are presented in Table 3. 

There were no histological differences between the excluded cases that had BRAF^V600E^ mutation as compared to the five excluded cases that did not have a BRAF^*V600E*^ mutation. 

**Figure 1:**
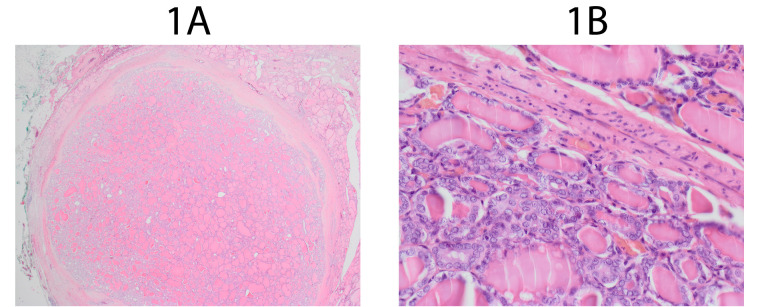
**A**. NIFTP 2018 at 12,5x magnification. **B**. NIFTP 2018 at 100x magnification.

**Figure 2:**
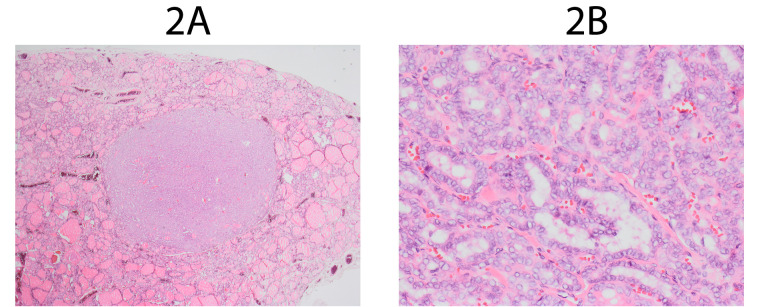
**A**. NIFTP 2016 at 12,5x magnification. **B**. NIFTP 2016 at 100x magnification.

Figures 1A and 1B show encapsulated NIFTP which meets revised criteria at 12,5x and 100x microscope magnification. Figures 2A and 2B show encapsulated tumor which meets NIFTP 2016 criteria and doesn’t meet NIFTP 2018 criteria. A single papillae can be spotted at 100x microscope magnification (pointed by arrow). 

Characteristics of allele mutation frequency in each NIFTP case is available as supplementary material. 

## DISCUSSION

**Table 1:** Table 1: Characteristics of patients with papillary thyroid cancer - AJCC 8^th^ Edition.

Feature	Total n = 1117 (100%)
Female, n (%)	983 (88%)
Male, n (%)	134 (12%)
Age at diagnosis (years) mean ± SD	51.3 ± 16.5
Female	50.1 ± 17.0
Male	56.0 ± 14.2
Tumor size (mm), mean ± SD (range)	13.2 ± 14.9 (0.5–130)
Tumor stage, n (%)	
T1	902 (80.7%)
T2	97 (8.7%)
T3	101 (9.1%)
T4	17 (1.5%)
Papillary cancer histologic subtype, n (%)	
Classic	920 (82.4%)
Follicular Other non-aggressive	173 (15.5%) 9 (0.8%)
Other aggressive	15 (1.3%)
Extrathyroidal extension, n (%)	
Negative	904 (80.9%)
Microscopic	180 (16.1%)
Gross	33 (3%)
Vascular invasion, n (%)	
Yes	66 (5.9%)
No	1051 (94.1%)
Multifocality, n (%)	
Yes	240 (21,5%)
No	877 (78,5%)
Lymph node metastasis, n (%)	
N0a	455 (40.7%)
N0b	532 (47.6%)
N1	130 (11.6%)
Distant metastasis, n (%)	
Yes	20 (1.8%)
No	1097 (98.2%)
TNM stage, n (%)	
I	1013 (90.7%)
II	72 (6.6%)
III	11 (1%)
IV	21 (1.9%)
ATA Initial Risk Stratification System, n (%)	
Low	771 (69%)
Intermediate High	295 (26.4%) 51 (4.6%)
Follow-up (years), median (range)	7.7 (1–16)
Status at final follow-up, n (%)	
NED	1058 (94.7%)
Biochemically persistent disease	39 (3.5%)
Structurally persistent disease	20 (1.8%)
Death, n (%)	20 (1.8%)

AJCC, American Joint Committee on Cancer; SD, standard deviation; ATA, American Thyroid Association; NED, no evidence of disease.

The definition of the new category of thyroid tumor, NIFTP, aimed to reduce the use of aggressive treatment in patients with indolent tumors. Initially, NIFTP was estimated to constitute as many as 18.6% of cases with PTC, which meant that the introduction of this new category would affect the therapeutic decisions concerning approximately 45,000 patients annually worldwide [5]. Subsequent researches estimated the proportion of PTC cases with NIFTP as 15–29% [7, 15, 16, 17]. Finally, a rate of 9.1% was agreed [9]; as this is based on application of the initial diagnostic criteria, without genetic examination of each case histopathologically diagnosed with NIFTP, it may still represent an overestimate [9]. In our research in a Polish population, the number of cases with NIFTP, diagnosed according to the revised criteria, was only 1.16% of all thyroid cancer cases. These data correspond with findings published by Parente et al. and Bychkov et al., where the percentages of cases with NIFTP were 2.1% and 1.6% in Canadian and Asian populations, respectively [9, 18]. Parente et al. applied restrictive diagnostic criteria, where only tumors without any papillae were diagnosed as NIFTP neoplasms, consistent with the up-to-date criteria [18]. The low frequency of NIFTP cases in Asian populations, reported by Bychkov et al., may result from a higher tendency to monitor for thyroid gland nodules, and postpone surgical treatment of patients classified as III and IV category of fine-needle aspiration cytology (FNAC), according to the Bethesda System for Reporting Thyroid Cytology [9, 19]. The observed variations in the prevalence of NIFTP in various reports may stem from different approaches to diagnosis, as well as treatment of thyroid nodules, or a lack of opportunity to examine whole tumor capsule during histopathological examination, the application of varying diagnostic criteria, or the subjective character of nuclear score assessment. In addition, the originally high frequency (reaching 15–24.6%) of cases with NIFTP could be attributable to the relatively small study populations included in these investigations [7, 15, 16, 17]. 

**Table 2:** Table 2: Characteristics of patients with NIFTP according to initial (2016) and revised (2018) criteria.

Feature	NIFTP 2016	NIFTP 2018
Number of patients	n = 23 (2.06% of all thyroid carcinomas)	n = 13 (1.16% of all thyroid carcinomas)
Female	22 (95.65%)	12 (94.31%)
Male	1 (4.35%)	1 (7.69%)
Age at diagnosis (years), mean		
± SD	50.52 ± 147	48.85 ± 12.90
Female	50.59 ± 14.50	48.83 ± 13.48
Male	49 ± 0	49 ± 0
Tumor size (mm), mean ± SD (range)	8.67 ± 12.18 (1–50)	10.88 ± 14.93 (1–50)
Genetic mutations, n (%)		
*BRAF*^V600E^**	5 (21.74%)	0 (0%)
*BRAF*^T599delinsTT^**	1 (4,35%)	0 (0%)
*TERT*	0 (0%)	0 (0%)
*TP53*	4 (17,39%)	3 (23,08%)
*KRAS*	5 (21,74%)	3 (23,08%)
*HRAS*	2 (8,70%)	2 (15,38%)
*NRAS*	1 (4,35%)	1 (7,69%)
*KIT*	2 (8,70%)	2 (15,38%)
*APC*	2 (8,70%)	1 (7,69%)
*PTEN*	1 (4,35%)	1 (7,69%)
*SMAD4*	1 (4,35%)	1 (7,69%)
*SMARCB1*	1 (4,35%)	1 (7,69%)
*HNF1A*	1 (4,35%)	1 (7,69%)
*MET*	1 (4,35%)	1 (7,69%)
*ATM*	1 (4,35%)	1 (7,69%)
Lymph node metastases (%)	0%	0%
Distant metastases (%)	0%	0%
Synchronic thyroid carcinoma	9/23 (39.13%)	3/13 (23.08%)
Follow-up (years)		
Mean ± SD	8.95 ± 4.56	9.14 ± 4.66
Median (range)	8.09 (2.21–16.27)	7.99 (2.21–14.89)

NIFTP, noninvasive follicular thyroid neoplasm with papillary-like nuclear features; SD, standard deviation

It is important not to qualify tumors with a high potential for malignancy as NIFTP, as restriction of aggressive treatment and oncologic care may lead to delayed diagnosis of recurrence or distant metastases. After the reclassification of EFVPTC as NIFTP, there were reports of metastases to the regional lymph nodes or distant metastases from tumors meeting the NIFTP criteria [18, 20]. Additionally, the outcomes of molecular examinations demonstrated that mutations with high oncogenic potential were present in some NIFTP tumors, including BRAF^*V600E*^, which is associated with a higher risk of recurrence and metastases to the regional lymph nodes [21, 22, 23]. Lee et al. found that, among tumors originally classified as NIFTP, BRAFV600E mutations were present in five cases (23.8%), and in one of these five patients, metastases were detected in the regional lymph nodes [21]. Other studies have also confirmed the occurrence of regional lymph node metastases associated with tumors that met the original diagnostic criteria for NIFTP [20, 24]. Based on all of these reports, it became clear that the initial NIFTP criteria were inadequate. 

To avoid diagnostic mistakes, leading to inaccurate PTC diagnosis, in 2018 a group of experts suggested that new diagnostic criteria for NIFTP should be introduced, in which the presence of well-developed papillae in <1% of the tumor precluded classification of a tumor as NIFTP. Also, molecular analyses are worth consideration as additional criteria; they are helpful but not obligatory for confirming the diagnosis of NIFTP, as they can determine the presence of the BRAF^*V600E*^ mutation, as well as other high-risk mutations (e.g., of *TERT* or *TP53*), and if these mutations are present, the tumor can still be classified as NIFTP, but in these cases intensified search for invasive features and papillae should be triggered [10]. Our research demonstrates that the application of the restrictive 2018 criteria, which exclude all cases with any papillae from the NIFTP group, leads to a situation where tumors with the BRAF*V600E* mutation are not diagnosed as NIFTP, but tumors with no papillae, which meet NIFTP 2018 criteria still can carry high-risk mutations like *TP53*. 

**Table 3:** Table 3: Characteristics of patients excluded using the 2018 NIFTP criteria.

Feature	Total n = 10 (0.90% of all thyroid carcinomas)
Female	10 (100%)
Male	0 (0%)
Age at diagnosis (years), mean ± SD	52.70 ± 16.10
Female	52.70 ± 16.10
Male	n/a
Tumor size (mm), mean ± SD (range)	5.80 ± 6.48 (1–20)
Genetic mutations, n (%)	
*BRAF^V600E^*	5 (50%)
*BRAF^T599delinsTT^*	1 (10%)
*TP53*	1 (10%)
*KRAS*	2 (20%)
*APC*	1 (10%)
*TERT* mutation (%)	0%
Lymph node metastases (%)	0%
Distant metastases (%)	0%
Papillae (< 1%), n (%)	10 (100%)
*BRAF^V600E^*-positive and papillae-positive, n (%)	5 (50%)
*BRAF^V600E^*-negative and papillae-positive, n (%)	5 (50%)
*BRAF^V600E^*-positive and papillae-negative, n (%)	0 (0%)
Follow-up (years)	
Mean ± SD	9.14 ± 4.66
Median (range)	10.86 (2.21–14.89)

NIFTP, noninvasive follicular thyroid neoplasm with papillary-like nuclear features; SD, standard deviation.

A relationship between the presence of papillae and the BRAF*^V600E^* mutation was identified by Point du Jour et al., who reported that there were no BRAF^V600E^ mutations in EFVPTC tumors that did not have any papillae [25]. A dependency between BRAF^V600E^ mutations and presence of well-developed papillae has been also found by other researchers [20, 24, 26, 27]. 

*TP53* mutation occurs almost exclusively in aggressive thyroid cancer subtypes and it is very unlikely that it can be present in benign thyroid lesions [28, 29, 30, 31]. In one case of our research simultaneous coincidence of *HRAS* and *TP53* mutation was spotted, which is known as an unfavourable factor in thyroid cancer treatment [32]. Therefore, long-term follow-up procedures even in tumors which fulfill revised NIFTP criteria are still needed. Furthermore, Parente et al. described a single case of tumor metastasis to the lungs from NIFTP with no papillae [18]. 

A limitation of this study is the retrospective nature of the research and the relatively small number of cases diagnosed with NIFTP; however, it is notable that all cases were diagnosed in one of the leading health centers in Poland for treatment of thyroid carcinoma; therefore, these data represent a valuable contribution to the discussion of this type of tumor. 

## MATERIALS AND METHODS

### Patients and Controls 

All research procedures were approved of by the city of Kielce Bioethical Commission, affiliated with the Holycross Region Chamber of Physicians (the number of the approval: 6/2020). All living patients who qualified for the inclusion in the research program regarding genetic testing were provided with information about its nature and objectives, as well as its methods, and then expressed their conscious consent to participate in the study. All those participants gave written consent before taking part in the research. Consent to our retrospective analysis of all the patients was given by the mentioned Bioethical Commission. 

This retrospective analysis was conducted at the Holycross Region Oncologic Health Centre, Kielce, which is a reference oncologic center offering comprehensive treatment for patients with thyroid neoplasms. 

During the period 2000–2016, 1993 patients diagnosed with PTC were treated at the Endocrinology Clinic. In those years 1140 patients were operated on in a single reference surgical center and qualified for inclusion in the research program. Histopathological samples from 1117 patients were retrieved from the archive (those of 23 patients were not found) for repeat histopathological assessment for NIFTP. 

NIFTP smears on glass slides were transported to the Molecular Diagnosing Laboratory for molecular assessment. Cases diagnosed as NIFTP according to the 2016 criteria were tested using the NGS method assessing mutations in the 50 most frequently mutated genes in cancer. Patient medical data included sex, age at diagnosis, tumor size, histological variant, angioinvasion, out-of-thyroid infiltration, multi-focus image, metastases to lymph nodes, TNM, applied treatment, response to treatment, and status at the end of observation. Hence, it was possible to determine how often NIFTP would have been diagnosed using both the initial and revised criteria. Associations between pathomorphological features and the molecular status of NIFTP samples were also assessed. 

### Pathological evaluation 

During the analysis period, the procedures used for pathomorphological examination of thyroid tumors remained unchanged. All thyroidectomy specimens were weighed, and the dimensions of the right and left lobes and isthmus were recorded. The entire outer surface was inked and serial cross-sections through the thyroid gland were done. Size, color, consistency, cysts, necrosis, location (upper, lower, right, left), encapsulation or infiltration, and relationship to the capsule (intact or with invasion of the capsule) were recorded for each lesion. 

Color, consistency, contour, and calcifications of the remaining parenchyma were described, and any adjacent soft tissues were examined for composition, presence of lymph nodes, or extension of the tumor into the soft tissue. 

Two pathologists with experience in the diagnosis of thyroid conditions, JK (20 years of practice) and AP (5 years of practice), conducted an additional examination of the samples to diagnose NIFTP. The initial NIFTP criteria, introduced by Nikiforov [5], were applied, as follows: encapsulation or clearly demarcated follicular growth pattern with <1% papillae, no psammoma bodies, <30% solid/trabecular/insular growth pattern, nuclear score 2–3, no vascular or capsular invasion, no tumor necrosis, and negative for high mitotic activity. 

Then, another examination of the samples was performed using the revised 2018 NIFTP criteria, as follows [10]: encapsulation or clear demarcation from adjacent thyroid parenchyma, follicular growth pattern with no well-formed papillae, no psammoma bodies, <30% solid/trabecular/insular growth pattern, nuclear features of PTC (nuclear score 2–3), no vascular or capsular invasion, no tumor necrosis, and negative for high mitotic activity. Molecular tests are helpful, but not required, for NIFTP diagnosis. When obtained, BRAF^*V600E*^ or other BRAF*^V600E^*-like or other high-risk mutations (*TERT*, *TP53*) should be absent in NIFTP. 

Disagreement between the findings of the two pathologists was resolved by discussion and consensus. 

### DNA Isolation 

A pathologist marked the area containing tumor cells on a hematoxylin and eosin-stained slide. Then, the tumor tissue on a matched unstained slide was deparaffinized using xylene and alcohol. The pathologist-selected areas from unstained slides were transferred to tubes for DNA isolation, using the Maxwell® 16 FFPE Tissue LEV DNA Purification Kit, according to the manufacturer’s instructions (Promega, USA). The concentration of isolated DNA was measured using a NanoDrop spectrophotometer (Thermo Scientific™, USA). 

### Sanger sequencing of the TERT promoter region 

We amplified the *TERT* promoter sequence using the following PCR primers: hTERTf (5’-CAGCGCTGCCTGAAACTC-3’) and hTERTr (5’-GTCCTGCCCCTTCACCTT-3’). After purification of the PCR products, sequencing was performed using a BigDye Terminator v1.1 Cycle Sequencing kit (Life Technologies, USA) and an ABI 3130 Automatic Capillary DNA Sequencer (Applied Biosystems, USA). 

In each batch of patients samples tested, we also test the sample with the previously detected mutation (positive control) and the sample without mutation (negative control).

### Next generation sequencing, library preparation 

The DNA was diluted to 10 ng/µl. The libraries were prepared using the Ion AmpliSeq™ Cancer Hotspot Panel v2 Kit, Manual Library Preparation and the Ion Xpress Barcode Adapters Kit (Thermo Fisher Scientific), according to the manufacturer’s instructions (Thermo Fisher Scientific). Ion Am-pliSeq™ Cancer Hotspot Panel v2 Kit (Thermo Fisher Scientific, USA), which allows the study of hotspots of 50 tumor genes (*ABL1, EZH2, JAK3, PTEN, ACT1, FBXW7, IDH2, PTPN11, ALK, FGFR1, KDR, RB1, APC, FGFR2, KIT, RET, ATM, FGFR3, KRAS, SMAD4, BRAF, FLT3, MET, SMARCB1, CDH1, GNA11, MLH1, SMO, CDKN2A, GNAS, MPL, SRC, CSF1R, GNAQ, NOTCH1, STK11, CTNNB1, HNF1A, NPM1, TP53, EGFR, HRAS, NRAS, VHL, ERBB2, IDH1, PDGFR, ERBB4, JAK2, PIK3CA*). One PCR-multiplex reactions were performed for each of the samples tested. The resulting multiplex PCR products were subjected to partial enzymatic digestion to remove primer sequences. Next, adapters for multiplex PCR products were enzymatically attached using the Ion Xpress Barcode Adapters Kit (Thermo Fisher Scientific). One of the adapters contains barcodes that allow identification of sequences from a given patient among a mixture of libraries. The prepared libraries were cleaned using Agencourt AMPure XP (Beckman Coulter Genomics) according to the manufacturer’s instructions (Ion AmpliSeq Library Kit 2.0 - Thermo Fisher Scientific). 

### Preparation of clonally amplified template for sequencing - emulsion PCR (emPCR) for S5 us-ing IonChef 

The concentration of libraries was measured by quantitative PCR with real-time detection (qRT-PCR) using the Ion Library TaqMan™ Quantitation Kit (Thermo Fisher Scientific) on a Rotor-Gene Q instrument (Qiagen). Based on the values obtained with qRT-PCR, all prepared libraries were diluted to a concentration of 100pM. Then, with Ion Chef (Thermo Fisher Scientific) and Ion 520 & Ion 530 Kit-Chef and Ion 530™ Chip Kit (Thermo Fisher Scientific), emPCR was performed, enrichment and two 530 chips were loaded (16-24 samples per chip, cov x1000). 

### Sequencing 

Sequencing was performed on an Ion S5 Prime sequencer (Thermo Fisher Scientific). Sequencing was done according to the manufacturer’s instructions.

### Bioinformatic analysis

The raw data generated during sequencing was processed using the Torrent Server Suite 5.12-TSS (Thermo Scientific, USA). The obtained sequences were matched (mapped) to the reference sequence of the human genome (hg19). Searching for different variants (SNP, deletions, insertions) was carried out using the Variant Caller 5.12 program which is part of Torrent Server Suite 5.12. The following basic parameters of the variants were used: minimum allele frequency SNP = 0.01 / INDEL = 0.05, minimal quality = 10, minimal sequencing depth = 10. Variant Caller is compatible with the IGV genomic browser - Integrative Genomics Viewer (Broad Institute), which enables fast visualization of sophisticated variants. To annotate the detected variants with the TSS, the wANNOVAR software (http://wannovar.wglab.org) was used. Additionally, Torrent Server Suite 5.12 generated FASTQ files that were used for analysis using the CLC Biomedical Workbench 5.0 (QIAGEN). The basic parameters used in the analysis were for CLC: minimum allele frequency = 0.01, minimal quality = 10, minimal sequencing depth = 100. Detected mutations, SNP, insertions and deletions of the coding regions of the analyzed genes were filtered to detect pathogenic mutations by COSMIC base, dbSNP database (to discard hereditary polymorphisms) and population base of the 1000GENOMES project. Only variants with minimal 5% allelic frequency were reported. In the case of variant of unknown significance or conflicting results we have performed in silico analysis using Varsome (https://varsome.com/) which integrates useful algorithms and databases, frequency in the populations and literature [33]. 

### Statistical Analysis 

Basic statistics (mean, standard deviation) were determined for continuous variables (age, tumor size, years of follow-up). Percentages were determined for discrete and ordinal variables. 

## CONCLUSION

NIFTP is a very rare type of thyroid neoplasm. The application of restrictive, revised histological diagnostic criteria for NIFTP makes BRAF*^V600E^* molecular examination unnecessary, but the presence of other high-risk mutations cannot be ruled out. 

The possible presence of high-risk mutations in NIFTP should make genetic examination a necessary step in diagnostic process of these tumors. 

## SUPPLEMENTARY TABLE


