# Supplementary material for: Are molecular tests necessary to diagnose NIFTP?
Source: Genes Cancer. 2021 Mar 15;12:39–50. doi: 10.18632/genesandcancer.213 (PMC8045962; doi:10.18632/genesandcancer.213)
Supplement: Supplementary file 1 [file ganc-12-39-s001.pdf]

Supplementary Table. Characteristics of allele frequency of the identified mutations

Patients who fulfill NIFTP 2016 and NIFTP 2018 diagnostic criteria				
**Case number**	**Gene**	**Mutation position**	**AA change**	**Allele frequency (%)**
**1**	***HRAS***	**c.A182G**	**p.Q61R**	**13**
**2**	***TP53***	**c.G569A**	**p.C190Y**	**5**
	SMAD4	**G302A**	**p.W101X**	**8**
**3**	***NRAS***	**c.A182G**	**p.Q61R**	**42**
**4**	***HRAS***	**c.C100T**	**p.P34S**	**48**
	HNF1A	**c.G599A**	**p.R200Q**	**37**
	TP53	**c.C457T**	**p.Q153X**	**31**
**5**	***MET***	**c.A3758G**	**p.Y1253C**	**5**
**6**	***KIT***	**c.G1681A**	**p.G561R**	**4**
**7**	***KRAS***	**c.A182G**	**p.Q61R**	**4**
	APC	**c.C4348T**	**p.R1450X**	**11**
**8**	***TP53***	**c.G701T**	**p.R234L**	**4**
**9**	***KRAS***	**c.A182G**	**p.Q61R**	**8**
**10**	***ATM***	**c.A1052T**	**p.D351V**	**34**
**11**	***SMARCB1***	**c.G152A**	**p.W51X**	**6**
	KIT	**c.G1576A**	**p.V526I**	**34**
**12**	***PTEN***	**c.C1030T**	**p.Q344X**	**7**
**13**	***KRAS***	**c.G40A**	**p.V14I**	**4**
**Patients who fulfill only NIFTP 2016 diagnostic criteria**				
**Case number**	**Genetic mutations**	**Mutation position**	**AA change**	**Allele frequency (%)**
**1**	***BRAF***	**c.T1799A**	**p.V600E**	**4**
**2**	***BRAF***	**c.T1799A**	**p.V600E**	**6**
**3**	***BRAF***	**c.T1799A**	**p.V600E**	**7**
**4**	***BRAF***	**c.T1799A**	**p.V600E**	**35**
**5**	***BRAF***	**c.T1799A**	**p.V600E**	**39**
**6**	***BRAF***	**c.1796_1797insTAC**	**p.T599delinsTT**	**31**
**7**	***TP53***	**c.A619T**	**p.M207L**	**10**
**8**	***APC***	**c.C4073T**	**p.A1358V**	**50**
**9**	***KRAS***	**c.A182G**	**p.Q61R**	**4**
**10**	***KRAS***	**c.G34C**	**p.G12R**	**12**

NIFTP, noninvasive follicular thyroid neoplasm with papillary-like nuclear features; AA, amino acids
